# Predictors of liver-related death among people who inject drugs in Vancouver, Canada: a 15-year prospective cohort study

**DOI:** 10.7448/IAS.17.1.19296

**Published:** 2014-11-10

**Authors:** Kanna Hayashi, Michael-John Milloy, Evan Wood, Huiru Dong, Julio SG Montaner, Thomas Kerr

**Affiliations:** 1 British Columbia Centre for Excellence in HIV/AIDS, Vancouver, Canada; 2 Division of AIDS, Faculty of Medicine, University of British Columbia, Vancouver, Canada; 3 Department of Family Practice, Faculty of Medicine, University of British Columbia, Vancouver, Canada

**Keywords:** injection drug use, hepatitis C virus infection, mortality, Canada

## Abstract

**Introduction:**

While HIV/AIDS remains an important cause of death among people who inject drugs (PWID), the potential mortality burden attributable to hepatitis C virus (HCV) infection among this population is of increasing concern. Therefore, we sought to identify trends in and predictors of liver-related mortality among PWID.

**Methods:**

Data were derived from prospective cohorts of PWID in Vancouver, Canada, between 1996 and 2011. Cohort data were linked to the provincial vital statistics database to ascertain mortality rates and causes of death. Multivariate Cox proportional hazards regression was used to examine the relationship between HCV infection and time to liver-related death. A sub-analysis examined the effect of HIV/HCV co-infection.

**Results and discussion:**

In total, 2,279 PWID participated in this study, with 1,921 (84.3%) having seroconverted to anti-HCV prior to baseline assessments and 124 (5.4%) during follow-up. The liver-related mortality rate was 2.1 (95% confidence interval [CI]: 1.5–3.0) deaths per 1,000 person-years and was stable over time. In multivariate analyses, HCV seropositivity was not significantly associated with liver-related mortality (adjusted relative hazard [ARH]: 0.45; 95% CI: 0.15–1.37), but HIV seropositivity was (ARH: 2.67; 95% CI: 1.27–5.63). In sub-analysis, HIV/HCV co-infection had a 2.53 (95% CI: 1.18–5.46) times hazard of liver-related death compared with HCV mono-infection.

**Conclusions:**

In this study, HCV seropositivity did not predict liver-related mortality while HIV seropositivity did. The findings highlight the critical role of HIV mono- and co-infection rather than HCV infection in contributing to liver-related mortality among PWID in this setting.

## Introduction

People who inject drugs (PWID) are at elevated risk of HIV and hepatitis C virus (HCV) infection [1, 2]. While HIV/AIDS remains one of the primary causes of death among this population worldwide [3], a recent study from Australia reported an increasing mortality burden of liver disease among opioid users [4]. However, little is known about trends of liver-related mortality among PWID in many settings. Further, although epidemics of viral hepatitis among PWID are presumed to contribute to elevated liver-related mortality in this population [2, 4], few studies have examined an independent contribution of HCV infection to liver-related deaths among PWID. While a recent study has identified a significant contribution of chronic HCV infection to liver-related deaths among Norwegian PWID aged >50 years [5], the contribution of other risk factors, including HIV infection [6] and alcohol use [4, 7], has not been fully examined.

Vancouver, Canada, has experienced an explosive HIV epidemic among PWID [8]. The estimated HCV prevalence in this population is also very high at >80% [9]. While previous literature indicates an increasing coverage of highly active antiretroviral therapy (HAART) among HIV-positive PWID and declining HIV incidence rates among PWID in this setting [10, 11], HCV treatment coverage remains very low at <10% [12]. This has led to increasing concerns about the potential mortality burden attributable to HCV infection among PWID. Therefore, we sought to examine the trend of liver-related deaths and the relationship between HCV infection and liver-related death among PWID in Vancouver.

## Methods

We pooled participants being followed in two well-characterized, on-going open prospective cohorts of drug users in Vancouver since 1996: the Vancouver Injection Drug Users Study (VIDUS) and the AIDS Care Cohort to Evaluate Access to Survival Services (ACCESS). The cohorts have been described in detail elsewhere [8, 13]. Briefly, VIDUS is a cohort of HIV-seronegative adult PWID who have injected an illicit drug in the month prior to baseline assessments. ACCESS is a cohort of HIV-seropositive adult drug users who have used an illicit drug other than cannabinoids in the previous month at the baseline interview. The two studies employed harmonized recruitment, primarily through snowball sampling and street outreach, and data collection tools. At baseline and semi-annually thereafter, participants answered an interviewer-administered questionnaire, which elicited data on demographic characteristics, drug-using behaviours and related exposures, and underwent serologic testing for HIV and HCV antibodies. Participants received $20 CAD for each study visit. The University of British Columbia/Providence Healthcare Research Ethics Board approved both studies.

We ascertained mortality rates and underlying causes of death among cohort participants through a confidential record linkage with the British Columbia Vital Statistics Agency and through on-going follow-up with contacts provided by participants. The specific methods employed have been described in detail elsewhere [14]. Briefly, all residents in the province of British Columbia have a unique and persistent government-provided identifier, which allows us to perform a semi-annual record linkage to the provincial Vital Statistics database with accuracy. In addition, on-going follow-up with contacts provided by participants have informed us of potential cases of death, for which we reviewed the registry data. The Vital Statistics database recorded causes of death according to the International Classification of Diseases, 10th edition (ICD-10).

Participants were eligible for the present study if they were recruited between 1 May 1996 and 31 December 2011 and had completed at least one follow-up visit during the study period. The sample was further restricted to individuals who reported having injected drugs in the previous six months at baseline. To avoid potential bias due to long durations between the last study visit and the date of death, individuals who were deceased more than 24 months after the last follow-up visit were censored on the last follow-up date.

The primary endpoint in this analysis was liver-related death, defined as having any of the following ICD-10 codes: viral hepatitis (B15–19), sequelae of viral hepatitis (B942), liver cancer (C22), alcoholic liver disease (K70) and non-alcoholic liver disease (K71–77). For an unspecified cause of death (ICD-10: R99), we referred to the community follow-up record for any indications of a liver-related death. The primary explanatory variable of interest was HCV serostatus (positive vs. negative), ascertained at baseline and via semi-annual serologic testing, and was treated as a time-updated variable. Our study protocol did not include HCV RNA testing. We also considered a range of secondary explanatory variables that might confound the relationship between HCV infection and time to liver-related death. Time-invariant variables assessed at baseline included: gender, ethnicity and time since first injection. As we were unable to ascertain timing of HCV infection, the time since first injection was included in the analysis as a surrogate measure [5]. Time-updated variables included age, HIV seropositivity and other behavioural variables that referred to the previous six months: unstable housing (i.e., living in single-room occupancy hotels, shelters, other transitional housing, or on the street); daily heroin injection; daily cocaine injection; and daily crack smoking; alcohol consumption (>4 drinks per day on average vs.≤4 drinks per day on average); engagement in sex work; and enrolment in methadone maintenance therapy. The cumulative number of incarceration events was also included as a time-updated variable. Variable definitions were consistent with those in our previous studies [9, 13, 15].

First, we examined baseline characteristics of the sample using descriptive statistics. Overall mortality rate and a 95% confidence interval [CI] during the study period were calculated using Poisson distribution. Liver-related mortality rates were calculated for the entire study period as well as at four-year intervals. Further, we identified an all-cause mortality rate and causes of death among HCV-seropositive participants to assess competing mortality risk in this population. Then, we used Cox proportional hazards regression to examine bivariate associations between each explanatory variable and the time to liver-related death. To fit the multivariate model, we employed a conservative stepwise backward selection approach. We included all variables found to be significantly associated with time to liver-related death in bivariate analyses at *p*<0.10 in a multivariate model and used a stepwise approach to fit a series of reduced models. After comparing the value of the coefficient associated with HCV serostatus in the full model to the value of the coefficient in each of the reduced models, we dropped the secondary variable associated with the smallest relative change. We continued this iterative process until the minimum change exceeded 5%. Remaining variables were considered as potential confounders in a final multivariate model.

We also conducted a sub-analysis to examine the effect of HIV/HCV co-infection on the time to liver-related death compared with HCV mono-infection. Here, we combined the HCV and HIV serostatus variables into a four-categorical variable: HCV-seropositive and HIV-seropositive, HCV-seronegative and HIV-seropositive, HCV-seronegative and HIV-seronegative, and HCV-seropositive and HIV-seronegative (our reference category). We fitted a multivariate Cox proportional hazards regression model including this new variable and the same set of covariates included in the final multivariate model in the primary analysis. All *p*-values were two-sided. All statistical analyses were performed using SAS software version 9.3 (SAS, Cary, NC).

## Results

In total, 2,279 PWID were recruited into this study and followed for a median of 60.9 months (interquartile range [IQR]: 34.4–113.2). Only one individual was right-censored due to a long duration (more than 24 months) between the last study visit and the date of liver-related death. Table 1 shows that 1,519 (66.7%) participants were male, and the median age at baseline was 37 years (IQR: 29–44). The median time since first injection at baseline was 14 years (IQR: 6–24). In total, 1,921 (84.3%) had seroconverted to anti-HCV prior to baseline assessments, and 124 (5.4%) additionally seroconverted during follow-up.

**Table 1:** Baseline characteristics of PWID participating in the VIDUS and ACCESS cohorts in Vancouver, Canada, between May 1996 and December 2011 (*n=*2,279)

Characteristic	*n* (%)
Age (median, IQR)	37 (29–44)
Male gender	1,519 (66.7%)
Caucasian ethnicity	1,393 (61.1%)
Years since first injection (median, IQR)	14 (6–24)
Unstable housing^a^	1,602 (70.3%)
Daily heroin injection^a^	881 (38.7%)
Daily cocaine injection^a^	711 (31.2%)
Daily crack smoking^a^	545 (23.9%)
Alcohol consumption (>4 drinks per day on average)^a^	366 (16.1%)
Engagement in sex work^a^	537 (23.6%)
Enrolment in methadone maintenance therapy^a^	514 (22.6%)
HIV seropositivity	620 (27.2%)
HCV seropositivity	1,921 (84.3%)

PWID: people who inject drugs; VIDUS: Vancouver Injection Drug Users Study; ACCESS: AIDS Care Cohort to Evaluate Access to Survival Services; IQR: interquartile range.

a Denotes activities during the six months prior to the interview.

**Figure 1:**
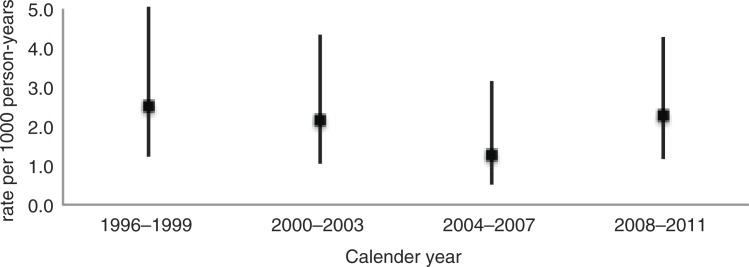
Liver-related mortality rates and 95% confidence intervals among PWID in Vancouver, Canada, 1996–2011.

In total, there were 487 deaths during the study period, with an overall mortality rate of 33.0 (95% CI: 30.0–36.1) deaths per 1,000 person-years. A total of 31 liver-related deaths were identified, yielding a liver-related mortality rate of 2.1 (95% CI: 1.5–3.0) deaths per 1,000 person-years. As shown in Figure 1, the rates at four-year intervals were relatively stable over time: 2.5 (95% CI: 1.3–5.0) in 1996–1999; 2.1 (95% CI: 1.1–4.3) in 2000–2003; 1.3 (95% CI: 0.5–3.1) in 2004–2007; and 2.3 (95% CI: 1.2–4.2) in 2008–2011. The primary underlying causes of liver-related death were viral hepatitis (*n*=12; 39%), non-alcoholic liver disease (*n*=8; 26%) and liver cancer (*n*=8; 26%). The mortality rates (per 1,000 person-years) due to viral hepatitis and non-alcoholic liver disease at four-year intervals appeared to have slightly decreased over time: 1.3 (95% CI: 0.5–3.3) and 1.3 (95% CI: 0.5–3.3), respectively, in 1996–1999; 0.8 (95% CI: 0.3–2.5) and 0.5 (0.1–2.1) in 2000–2003; 0.8 (95% CI: 0.3–2.4) and 0.3 (95% CI: 0.04–1.8) in 2004–2007; and 0.5 (95% CI: 0.1–1.8) and 0.2 (95% CI: 0.03–1.6) in 2008–2011. In contrast, the mortality rates due to liver cancer appeared to have increased over time: 0 death in 1996–1999 and 2000–2003; 0.3 (95% CI: 0.04–1.8) in 2004–2007; and 1.6 (95% CI: 0.8–3.4) in 2008–2011.

Among HCV-seropositive participants (*n*=2045), there were 465 deaths, and an all-cause mortality rate was 34.2 (95% CI: 31.3–37.4) deaths per 1,000 person-years. Common causes of death among this sample included HIV-related (24.7%), other non-accidental causes (e.g., respiratory and circulatory diseases, neoplasmas; 24.1%), overdose (23.7%), suicide and other accidental causes (12.3%), and liver related (6.0%).

Table 2 shows the results of bivariate and multivariate Cox proportional hazards regression analyses. As shown, in multivariate analysis, HCV seropositivity was not significantly associated with liver-related mortality (adjusted relative hazard [ARH]: 0.45; 95% CI: 0.15–1.37), but HIV seropositivity was independently and positively associated with the outcome (ARH: 2.67; 95% CI: 1.27–5.63). In sub-analysis, individuals with HIV/HCV co-infection had a 2.53 (95% CI: 1.18–5.46) times hazard of liver-related death compared with those with HCV mono-infection.

**Table 2:** Bivariate and multivariate Cox proportional hazards regression analysis of factors associated with liver-related mortality among PWID in Vancouver, Canada (*n*=2,279)

	Relative hazard (RH)	
	
Characteristic	Unadjusted (95% CI)	Adjusted (95% CI)
Age^b^		
Per 10 years older	1.95 (1.36–2.80)	1.59 (0.90–2.82)
Gender		
Male vs. female	3.01 (1.18–7.66)	2.38 (0.86–6.58)
Ethnicity		
Caucasian vs. Others	1.80 (0.82–3.96)	
Time since first injection		
Per year longer	1.05 (1.01–1.09)	1.02 (0.98–1.07)
Unstable housing^a^^,^ b		
Yes vs. no	1.12 (0.55–2.29)	
Heroin injection^a^^,^ b		
Daily vs.<daily	0.62 (0.24–1.61)	
Cocaine injection^a^^,^ b		
Daily vs.<daily	1.10 (0.43–2.82)	
Crack smoking^a^^,^ b		
Daily vs.<daily	0.51 (0.20–1.34)	
Alcohol consumption^a^^,^ b		
>4 drinks per day vs. ≤4 drinks per day	1.11 (0.32–3.90)	
Engagement in sex work^a^^,^ b		
Yes vs. no	0.25 (0.03–1.76)	
Enrolment in methadone maintenance therapy^a^^,^ b		
Yes vs. no	0.84 (0.42–1.71)	
Incarceration events^b^		
1–2 times vs. never	1.03 (0.45–2.32)	
3–5 times vs. never	0.68 (0.22–2.05)	
>5 times vs. never	0.74 (0.19–2.83)	
HIV serostatus^b^		
Positive vs. negative	2.17 (1.06–4.43)	2.67 (1.27–5.63)
HCV serostatus^b^		
Positive vs. negative	0.85 (0.26–2.77)	0.45 (0.15–1.37)

PWID: people who inject drugs; CI: confidence interval.

a Refers to activities during the six months prior to interview.

b Denotes time-updated variables.

## Discussion

Our results demonstrate that liver-related mortality rates among our cohorts of PWID in Vancouver were stable between 1996 and 2011. We also found that HIV seropositivity rather than HCV seropositivity predicted liver-related mortality. Further, we found that HIV/HCV co-infection had a significantly higher risk of liver-related mortality than HCV mono-infection.

The liver-related mortality rate identified in our study was slightly higher than a pooled liver-related mortality rate reported in a previous meta-analysis [16]. However, unlike the previous report from Australia [4], our findings did not indicate an increasing trend of liver-related death over time. Given a very low uptake of HCV treatment in our setting [12], it is unlikely that the trend reflects potential benefits of therapeutic interventions for HCV. A more plausible explanation for the discrepancy may be that competing mortality risk was higher in our study population compared with that in Australia, which was drawn from a registry of patients receiving opioid substitution treatment [4]. Further, the low HIV prevalence among Australian PWID (<2%), compared to our setting (>15%), may also explain the discrepancy [17, 18]. High competing mortality risk among our sample was also reflected in the overall mortality rate of 33.0 deaths per 1,000 person-years, which was significantly higher than a previously reported pooled mortality rate of 23.5 deaths per 1,000 person years among PWID worldwide [3]. The low liver-related mortality rate among our sample may also reflect the relatively short follow-up time since HCV infection in our study (an estimated 19 years on average). Given that disease progression in chronic HCV would take as long as >15 to 20 years [19–21] , and considering the increasing rates of death from liver cancer observed in our study, increases in liver-related mortality may follow in this setting. Continued monitoring of rates of liver-related morbidity and mortality among PWID, particularly among individuals infected with HCV at younger age, is needed in this setting.

We found that HIV infection, rather than HCV infection, was an independent predictor of liver-related death. This finding was congruent with recent studies reporting increasing proportions of liver-related mortality among people living with HIV/AIDS [6, 22]. Our finding that individuals with HIV/HCV co-infection had a greater risk of liver-related mortality than those with HCV mono-infection was also consistent with a meta-analysis showing a higher rate of cirrhosis in HIV/HCV co-infection compared with HCV mono-infection [23]. The meta-analysis further suggested that HAART did not improve the adverse effect of HIV infection on HCV prognosis [23]. Collectively, these findings highlight the importance of promoting access to diagnostics and treatment for liver disease among HIV-positive PWID.

Our study has several limitations. First, as the study sample was not randomly selected, our findings may not be generalizable to other populations of PWID. Second, the self-reported data may be affected by reporting biases, including recall bias and socially desirable responding. However, we note that this type of data has been commonly utilized in observational studies involving PWID and found to be valid [24]. Third, as with all observational studies, the relationships between the explanatory variables and outcome assessed may be under the influence of unobserved confounding. While we sought to address this bias with multivariate adjustment of the key demographic and behavioural predictors of survival, residual confounding may include participants’ clinical characteristics, including duration of HCV infection, our reliance on antibody rather than RNA status (e.g., spontaneous HCV clearance was not examined), access to HIV and/or HCV treatment, and treatment outcomes. Lastly, the liver-related mortality rate reported herein may be underestimated due to potential biases associated with the use of the death registry data. For example, approximately 10% of deaths had unspecified causes. As the registry only records a primary cause of death, liver disease might have been underrepresented in the presence of another condition or injury that was more directly responsible for the death [25]. Further, deaths occurring outside of the province were not recorded in the registry, although we note that migration rates among PWID have been shown relatively low in this setting [26]. The low event count resulted in wide intervals around some of the estimates reported and made it difficult to undertake additional analyses (e.g., to examine the impact of HAART on liver-related mortality among HIV-positive PWID).

## Conclusions

In summary, HIV seropositivity rather than HCV seropositivity predicted liver-related mortality among our cohorts of PWID in Vancouver. The findings highlight the role of HIV mono- and co-infection with HCV rather than HCV infection in contributing to liver-related mortality among PWID. Our results highlight the need to expand efforts to promote access to HCV testing and treatment, particularly among HIV-positive PWID.
